# Lack of association between heart period variability asymmetry and respiratory sinus arrhythmia in healthy and chronic heart failure individuals

**DOI:** 10.1371/journal.pone.0247145

**Published:** 2021-02-16

**Authors:** Beatrice De Maria, Laura Adelaide Dalla Vecchia, Roberto Maestri, Gian Domenico Pinna, Monica Parati, Francesca Perego, Vlasta Bari, Beatrice Cairo, Francesca Gelpi, Maria Teresa La Rovere, Alberto Porta

**Affiliations:** 1 IRCCS Istituti Clinici Scientifici Maugeri, Milan, Italy; 2 IRCCS Istituti Clinici Scientifici Maugeri, Montescano, Pavia, Italy; 3 Neuroengineering and Medical Robotics Laboratory, Department of Electronics, Information and Bioengineering, Politecnico di Milano, Milan, Italy; 4 Department of Cardiothoracic, Vascular Anesthesia and Intensive Care, IRCCS Policlinico San Donato, San Donato Milanese, Milan, Italy; 5 Department of Biomedical Sciences for Health, University of Milan, Milan, Italy; Sahlgrenska University Hospital, SWEDEN

## Abstract

Temporal asymmetry is a peculiar aspect of heart period (HP) variability (HPV). HPV asymmetry (HPVA) is reduced with aging and pathology, but its origin is not fully elucidated. Given the impact of respiration on HPV resulting in the respiratory sinus arrhythmia (RSA) and the asymmetric shape of the respiratory pattern, a possible link between HPVA and RSA might be expected. In this study we tested the hypothesis that HPVA is significantly associated with RSA and asymmetry of the respiratory rhythm. We studied 42 middle-aged healthy (H) subjects, and 56 chronic heart failure (CHF) patients of whom 26 assigned to the New York Heart Association (NYHA) class II (CHF-II) and 30 to NYHA class III (CHF-III). Electrocardiogram and lung volume were monitored for 8 minutes during spontaneous breathing (SB) and controlled breathing (CB) at 15 breaths/minute. The ratio of inspiratory (INSP) to expiratory (EXP) phases, namely the I/E ratio, and RSA were calculated. HPVA was estimated as the percentage of negative HP variations, traditionally measured via the Porta’s index (PI). Departures of PI from 50% indicated HPVA and its significance was tested via surrogate data. We found that RSA increased during CB and I/E ratio was smaller than 1 in all groups and experimental conditions. In H subjects the PI was about 50% during SB and it increased significantly during CB. In both CHF-II and CHF-III groups the PI was about 50% during SB and remained unmodified during CB. The PI was uncorrelated with RSA and I/E ratio regardless of the experimental condition and group. Pooling together data of different experimental conditions did not affect conclusions. Therefore, we conclude that the HPVA cannot be explained by RSA and/or I/E ratio, thus representing a peculiar feature of the cardiac control that can be aroused in middle-aged H individuals via CB.

## Introduction

Heart period (HP) varies on a beat-to-beat basis and this dynamic is denoted as HP variability (HPV). HPV features an imbalance between negative (i.e. heart rate accelerations) and positive (i.e. heart rate decelerations) variations in young healthy subjects and this phenomenon is labelled as HPV asymmetry (HPVA). More specifically, HPVA leads to runs of HP lengthening shorter than those of HP shortening [1, 2]. HPVA can be quantified from HPV recordings via the Porta’s index (PI) computing the percentage of negative HP variations [3]. When PI is above 50%, the greater the PI, the more important the HPVA. The relevance of HPVA lies in being a feature observed with an intact cardiac neural control and in being absent in pathological conditions. In healthy subjects HPVA is enhanced during passive and active orthostatic challenges [2–7] and during daytime [8]. It is linked to the autonomic nervous system development in the fetus [5, 9], it is reduced with aging [7, 10, 11] and it is disrupted by many pathological conditions [8, 10–13]. Since the presence of HPVA makes statistical properties of HPV series different under time reversal [2–5, 8, 10, 11] and this feature is not observable in linear processes [14], HPVA is considered to be one of the determinants of the nonlinear dynamics in short-term HPV. The link of HPVA with nonlinear HPV dynamics makes HPVA assessment the typical target of those HPV studies checking for the presence of nonlinear HPV dynamics as a hallmark of healthy cardiac control [10, 15, 16]. In spite of the numerous studies carried out for its characterization, the physiological mechanism responsible for HPVA remains unclear. Among the possible mechanisms, the peripheral hypothesis supports a strong involvement of the cardiac baroreflex, namely the reflex that aims at adjusting HP in response to arterial pressure changes. Since the baroreflex exhibits an asymmetric behavior that leads to greater HP variations in response to arterial pressure rises than falls, it has been suggested that this peculiar property might explain HPVA [7]. However, this peripheral hypothesis does not exclude the concurrent action of additional influences. Respiration has the potential of producing HPVA: indeed, it is an asymmetric input featuring an inspiratory (INSP) phase shorter than the expiratory (EXP) one with an INSP to EXP (I/E) ratio near to 1:2 [17, 18]. In addition, respiration is accompanied by HP fluctuations at the respiratory rate [19–22], known as respiratory sinus arrhythmia (RSA) [23], grouping tachycardic runs in INSP and bradycardic ones in EXP. Accordingly, it was found that HPVA increases with modifications of the I/E ratio from the more physiological value of 1:2 to 2:1 [18]. While keeping a physiological I/E ratio, it has been suggested [7] that, in presence of a negligible RSA (i.e. with limited HP variations at the respiratory rate), the PI is about 50% or even lower given that the INSP duration is shorter than the EXP one. Conversely, if the magnitude of RSA is remarkable, a migration of PI toward 50% and above this value is expected [7], thus imposing a significant and positive correlation between PI and RSA.

The aim of this study was to check the relation between HPVA and RSA in populations featuring a limited RSA and a physiological value of the I/E ratio smaller than 1, namely middle-aged healthy (H) subjects and chronic heart failure (CHF) patients, and to test the effect of a maneuver, namely controlled breathing (CB), empowering the RSA. The asymmetric behavior of respiration was quantified via the ratio of the INSP to EXP duration, i.e. the I/E ratio [17], the HPVA via the PI [2, 8] and the RSA as the power of HPV series at the respiratory rate [19–22]. We hypothesized that, in presence of a physiological value of the I/E ratio smaller than 1, HPVA could be significantly associated with RSA and this association could become more visible when RSA was increased via CB. Preliminary results were presented at the 11th meeting of the European Study Group of the Cardiovascular Oscillations [24].

## Methods

### Ethics statement

The study was in keeping with the Declaration of Helsinki. The study was approved by the ethical review board of the IRCCS Istituti Clinici Scientifici Maugeri (approval number: 211; approval date: 17/04/2002). Written signed informed consent was obtained from all subjects.

### Population and experimental protocol

We studied two groups of CHF patients assigned to class II (CHF-II) and III (CHF-III) according to classification criteria of the New York Heart Association and a group of age-matched H subjects. CHF-II and CHF-III groups had similar age and reduced left ventricular ejection fraction (LVEF). In detail, we considered: i) 42 H subjects (age: 60±5 yrs; 42 males); ii) 26 CHF-II patients (age: 57±4 yrs; LVEF: 27%±6%; 23 males); iii) 30 CHF-III (age: 58±5 yrs; LVEF: 28%±8%; 23 males). All the CHF-II and CHF-III patients were clinically stable, in sinus rhythm and had no recent (<6 months) myocardial infarction or cardiac surgery. Table 1 summarizes the demographic and clinical characteristics of CHF-II and CHF-III patients. A detailed clinical interview and physical examination excluded the presence of any sign or symptom of cardiac diseases in H subjects. None of them were taking any medication or had disturbances known to affect the autonomic nervous system. The H subjects were also in sinus rhythm.

**Table 1:** Demographic and clinical characteristics of CHF-II and CHF-III patients.

	CHF-II (n = 26)	CHF-III (n = 30)
Age [yrs]	57±4	58±5
Gender [males/females]	23/3	23/7
BMI [kg·m^-2^]	28±4	28±4
LVEF [%]	27±6	28±8
Coronary artery disease	11 (42)	20 (67)
Hypertensive cardiomyopathy	3 (12)	1 (3)
Valvular cardiomyopathy	1 (4)	0 (0)
Idiopathic cardiomyopathy	11 (42)	9 (30)
Hypertension	8 (31)	12 (40)
Diabetes	3 (11)	4 (30)
eGFR [ml·min^-1^]	76±17	68±19
Mild-to-moderate renal failure (eGFR<45 ml·min^-1^)	1 (4)	3 (10)
Moderate-to-severe renal failure (eGFR<30 ml·min^-1^)	0 (0)	1 (3)

NYHA: New York Heart Association; CHF: chronic heart failure; CHF-II: CHF in NYHA class II; CHF-III: CHF in NYHA class III; BMI: body mass index; LVEF, left ventricular ejection fraction; eGFR, estimated glomerular filtration rate. Continuous variables are presented as mean±standard deviation. Categorical variables as absolute numbers (percentage).

The experimental protocol was conducted at IRCCS Istituti Clinici Scientifici Maugeri, Montescano, Italy and was the same for the three different groups of individuals. The subjects were studied in the morning in supine position. After instrumentation, subjects carried out a session of familiarization with the paced breathing protocol. During this phase they were instructed to follow a recorded human voice indicating the onset of the INSP and EXP phases at a rate of 0.25 Hz with a I/E ratio of about 0.7. After an initial trial of two minutes, they were asked whether they felt comfortable with the CB pacing or would rather prefer to slightly increase or decrease it. Accordingly, an adjustment was made within ±10% of the target value (i.e. 0.25 Hz), while I/E ratio was preserved. After some minutes of stabilization, we acquired a surface electrocardiogram (ECG) with a ECG bioamplifier (Cardiolab3, Marazza, Monza, Italy) and lung volume (Respitrace, Florida, USA). The ECG device preserved frequencies between 0.01 and 125 Hz. Lung volume signal was low-pass filtered at 1 Hz. Signals were monitored for 8 minutes during spontaneous breathing (SB) and for 8 minutes during CB at 15 breaths/minute (i.e. 0.25 Hz). During CB subjects were guided according to the procedure utilized during the familiarization process and the same personalized rate. ECG and lung volume signals were sampled at 250 Hz. Due to the low quality of the respiratory signal in a fraction of subjects the reliable detection of respiratory phase onsets was carried out in 26 H, 26 CHF-II and 28 CHF-III subjects.

### Extraction of the beat-to-beat HP and breath-to-breath respiratory phase duration series

The HP was approximated as the temporal distance between two consecutive R-wave peaks on the ECG [25]. The R-wave was identified by an algorithm based on a threshold on the first derivative of the ECG. The R-wave apex location was fixed via parabolic interpolation.

Maxima and minima of the respiratory signal were detected by setting a confidence interval about the mean value of the respiratory signal [26]. The amplitude of the confidence interval was defined as a fraction of the difference between a maximum and a minimum chosen graphically over the respiratory signal by the user. Peaks and valleys outside this confidence interval were identified. Their time occurrences were taken as EXP and INSP onsets.

The detections of the R-wave peaks and INSP and EXP onsets were manually corrected in case of erroneous identification. Missing detections were manually inserted. HP series of 256 consecutive values were selected randomly in each experimental session (i.e. SB and CB). The HP mean was computed, labelled as μ_HP_ and expressed in ms. INSP and EXP onsets were extracted during the same period and the INSP and EXP durations were computed. INSP and EXP durations were expressed in s. The respiratory period was defined as the temporal distance between two EXP onsets. The ratio between INSP and EXP durations was calculated for each respiratory cycle and labeled I/E ratio. The I/E ratio is dimensionless. In physiological condition the I/E ratio is smaller than 1 [17, 18]. In this situation, the smaller the I/E ratio, the greater the respiratory signal asymmetry.

Computation of variability series and all the analyses described in the next subsections were carried out via signal processing and time series analysis programs developed in-house.

### Computation of the RSA

The RSA was estimated via parametric spectral analysis. Briefly, HPV series were described as a realization of an autoregressive (AR) process modeling the variation of the most recent HP about μ_HP_ as a linear combination of *p* past HP changes weighted by constant coefficients plus a sample drawn from a realization of a zero mean white noise, where *p* is the order of the AR model [27, 28]. The coefficients of the AR model and the variance of the white noise were identified directly from the series by solving the least squares problem via Levinson-Durbin recursion [27]. The number *p* of coefficients was chosen according to the Akaike’s figure of merit in the range from 8 to 16 [29]. Power spectral density was computed from the AR coefficients and from the variance of the white noise according to the maximum entropy spectral estimation approach [27]. The power spectral density was factorized into a sum of terms, referred to as spectral components, the sum of which provides the entire power spectral density [28]. Power spectral decomposition provided the central frequency of the components expressed in normalized frequency units, namely cycles per beat. Central frequency ranged from 0 to 0.5 cycles/beat and was converted into Hz by dividing the value by the average sampling period T = μ_HP_ expressed in s [28]. RSA was estimated as the sum of the powers of all the spectral components whose central frequencies dropped in the high frequency band (from 0.15 to 0.5 Hz) [19, 20]. RSA was expressed in ms^2^.

### Evaluation of the HPVA

The HPVA was quantified via the percentage of negative HP variations with respect to the total amount of HP changes via the PI [2, 3]. PI ranges from 0 to 100 and it is expressed in %. A PI>50% indicates the presence of an asymmetric behavior of the HP series with tachycardic runs longer than the bradycardic ones.

### Generation of the surrogate data

We verified the presence of asymmetry in the original HP series via a surrogate data approach. We created one artificial surrogate series for each subject in each experimental condition. The surrogate series was generated via the iterative amplitude-adjusted Fourier transform-based method [30, 31]. The surrogates perfectly preserved the distribution of values of the original series and their power spectral density was the best approximation of the power spectral density of the original series according to the number of iterations of the procedure (here 100). Conversely, any pattern of phases was destroyed by substituting the phases of the original series with numbers taken from a uniform distribution from 0 to 2π. The PI was computed over both the original and surrogate series. Since HPVA is not expected to be present in surrogates featuring only linear dynamics [14], when a significant difference between the PI computed over the original and that calculated over the surrogate series was found, we assumed that HPVA was present [2, 3, 5].

### Statistical analysis

Two-way repeated measures analysis of variance (one-factor repetition, Holm-Sidak test for multiple comparisons) was applied to assess the difference of μ_HP_, RSA and I/E ratio between experimental conditions (i.e. SB and CB) within the same group (i.e. H, CHF-II or CHF-III) and among different groups within the same experimental condition. Assigned the group, the same test (two-factor repetition) was exploited to check the difference of respiratory phase duration between types of respiratory phase (i.e. INSP and EXP) within the same experimental condition (i.e. SB or CB) and between experimental conditions within the same type of respiratory phase. Assigned the group, the same test (two-factor repetition) was utilized to verify the difference of PI between types of series (i.e. original and surrogate sequences) within the same experimental condition (i.e. SB or CB) and between experimental conditions within the same type of series. The associations between PI and I/E ratio and between PI and RSA were checked in each experimental condition and group via Pearson correlation analysis. The same association was also tested in each group by pooling the data together regardless of the experimental condition (i.e. SB and CB). Pearson product moment correlation coefficient *r* and type I error probability *p* were calculated. Data are presented as mean±standard deviation. Statistical analysis was carried out using the statistical program Sigmaplot (Sigmaplot, v.14.0, Systat Software, Inc., Chicago, IL, USA). A *p*<0.05 was always considered as significant.

## Results

**Fig 1:**
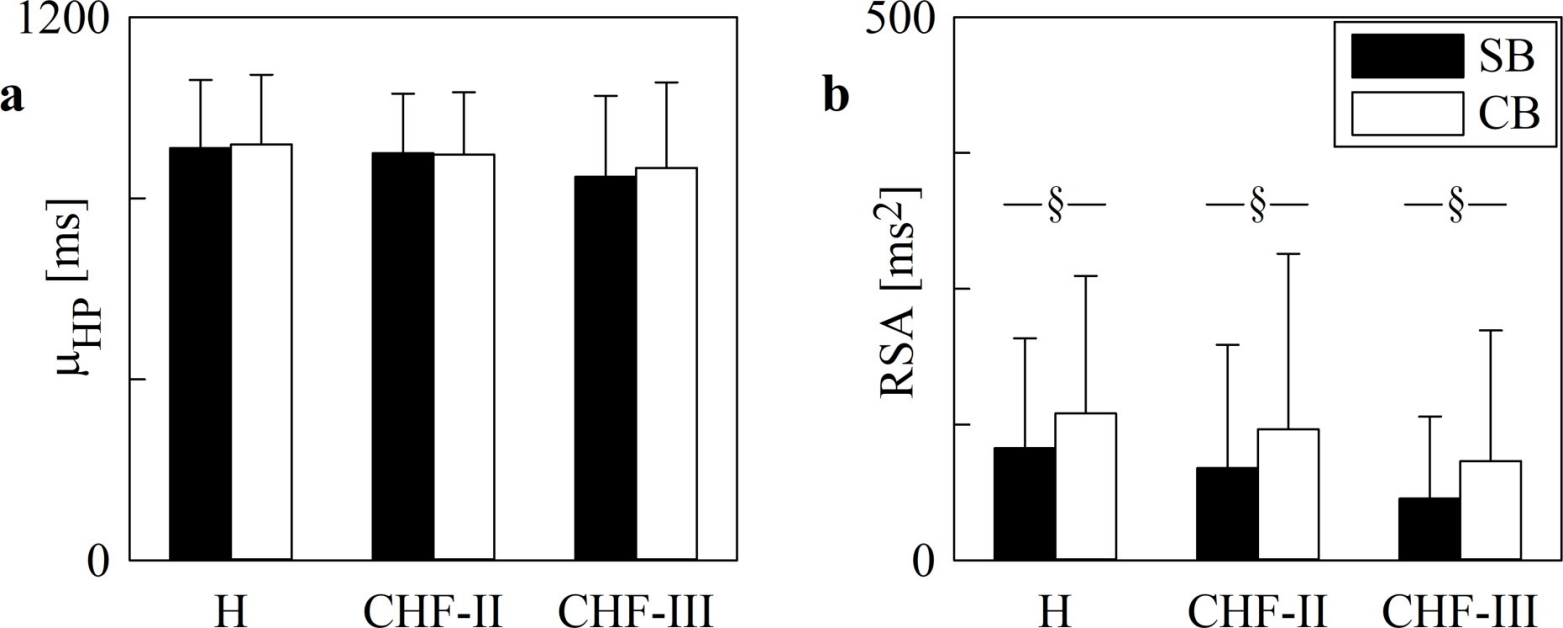
μ_HP_ and RSA in H, CHF-II, CHF-III groups during SB and CB. The grouped error bar graphs show μ_HP_ (a) and RSA (b) in H, CHF-II and CHF-III groups. The markers are computed during SB (solid black bars) and CB (open white bars). Data are reported as mean±standard deviation. The symbol § indicates *p*<0.05 between SB and CB within the same group.

The grouped error bar graphs of Fig 1 show μ_HP_ (Fig 1A) and RSA (Fig 1B) as a function of the group (i.e. H, CHF-II and CHF-III). Data were collected during SB (solid black bars) and CB (open white bars). The μ_HP_ did not vary with group and experimental condition. Regardless of the experimental condition, the RSA remained similar across groups. Conversely, CB increased RSA compared to SB in all the groups.

**Fig 2:**
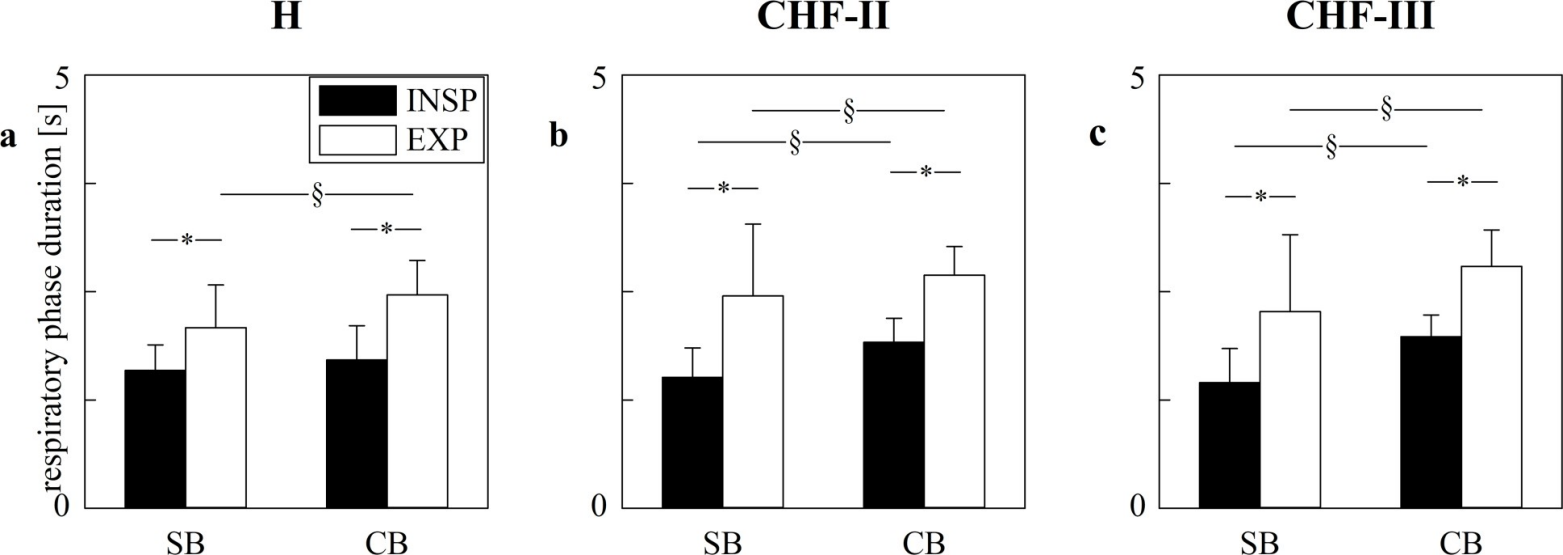
Respiratory phase durations in H, CHF-II, CHF-III groups during SB and CB. The grouped error bar graphs show INSP (solid black bars) and EXP (open white bars) durations as a function of the experimental condition (i.e. SB and CB). Respiratory phase durations are compared in H (a), CHF-II (b) and CHF-III (c) groups. Data are reported as mean±standard deviation. The symbol § indicates *p*<0.05 between SB and CB within the same respiratory phase. The symbol * indicates *p*<0.05 between INSP and EXP durations within the same experimental condition.

The grouped error bar graphs of Fig 2 show the duration of the respiratory phases as a function of the experimental condition (i.e. SB and CB). Data are relevant to the INSP (solid black bars) and EXP (open white bars). The graphs show the data collected in H (Fig 2A), CHF-II (Fig 2B) and CHF-III (Fig 2C) groups. Regardless of the group and experimental condition, EXP duration was longer than the INSP one. Both INSP and EXP durations increased during CB compared to SB and this result held in all groups with the exception of the H group in which the increase was observed solely in the EXP phase.

**Fig 3:**
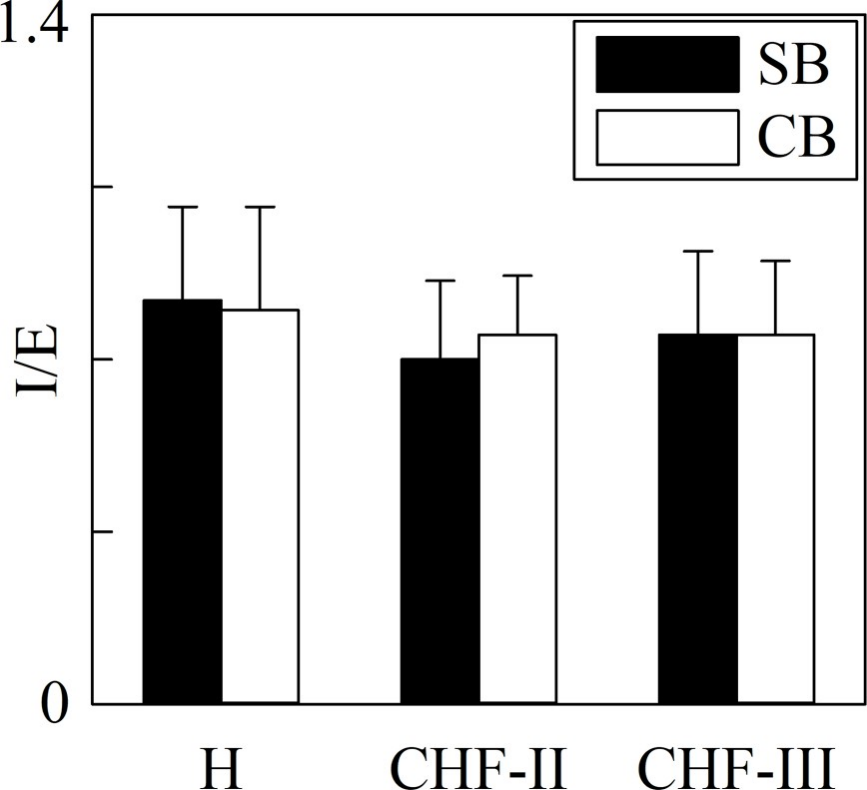
I/E ratio in H, CHF-II, CHF-III groups during SB and CB. The grouped error bar graph shows I/E ratio in H, CHF-II and CHF-III groups. The markers are computed during SB (solid black bars) and CB (open white bars). Data are reported as mean±standard deviation.

Fig 3 has the same structure as Fig 1 but it shows the I/E ratio. This respiratory marker remained unvaried across groups and experimental conditions.

**Fig 4:**
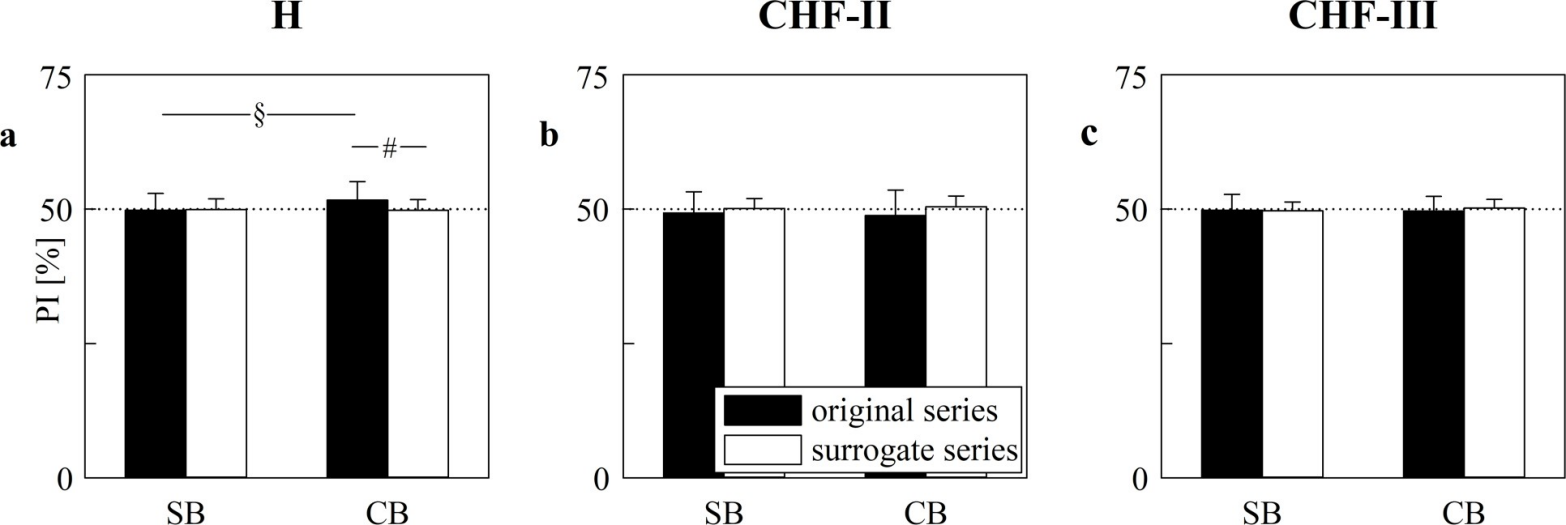
PI computed over original and surrogate HP series in H, CHF-II, CHF-III groups. The grouped error bar graphs show PI as a function of the experimental condition (i.e. SB and CB) in H (a), CHF-II (b) and CHF-III (c) groups. The PI is computed over original (solid black bars) and surrogate (open white bars) HP series. Data are reported as mean±standard deviation. The symbol § indicates *p*<0.05 between SB and CB within the same type of series. The symbol # indicates *p*<0.05 between original and surrogate HP series within the same experimental condition.

The grouped error bar graphs of Fig 4 show the PI computed over the original series (solid black bars) and surrogate ones (open white bars) as a function of the experimental condition (i.e. SB and CB). The grouped error bar graphs are relevant to the PI computed over H (Fig 4A), CHF-II (Fig 4B) and CHF-III (Fig 4C) groups. The PI calculated over the original data was significantly different from that computed over surrogates only during CB in H individuals. The PI calculated over the original data increased during CB compared to SB solely in H subjects, while CB did not affect PI in CHF patients. As expected the PI computed over the surrogate series remained close to 50% and constant across the experimental conditions and this result held regardless of the group.

Table 2 shows the results of the correlation analysis between PI and I/E ratio and between PI and RSA in each group and experimental condition. Regardless of the group and experimental condition, no significant correlation was found. Table 3 shows the results of the correlation analysis between PI and RSA and between PI and I/E ratio in each group when data were pooled together regardless of the experimental condition. Regardless of the group, we confirmed that no significant correlation was found.

**Table 2:** Results of the correlation analysis of PI with I/E ratio and RSA in H, CHF-II and CHF-III groups during SB and CB.

marker	H				CHF-II				CHF-III			
	SB		CB		SB		CB		SB		CB	
	*r*	*p*	*r*	*p*	*r*	*p*	*r*	*p*	*r*	*p*	*r*	*p*
I/E	-0.049	8.1×10^−1^	-0.261	1.4×10^−1^	0.220	2.8×10^−1^	0.187	3.6×10^−1^	0.034	8.6×10^−1^	-0.257	1.8×10^−1^
RSA [ms^2^]	-0.239	0.127	0.087	0.582	0.029	0.890	-0.029	0.886	-0.107	0.581	0.014	0.942

NYHA: New York Heart Association; H: healthy controls; CHF: chronic heart failure; CHF-II: CHF in NYHA class II; CHF-III: CHF in NYHA class III; SB: spontaneous breathing; CB: controlled breathing at 15 breaths per minute; I/E: ratio of the inspiratory phase duration to the expiratory one; RSA: respiratory sinus arrhythmia; *r*: Pearson product moment correlation coefficient; *p*: probability of type I error.

**Table 3:** Results of the correlation analysis of PI with I/E ratio and RSA in H, CHF-II and CHF-III groups after pooling together data relevant to SB and CB.

marker	H		CHF-II		CHF-III	
	*r*	*p*	*R*	*p*	*r*	*p*
I/E	-0.194	1.3×10^−1^	0.187	1.8×10^−1^	-0.156	2.4×10^−1^
RSA [ms^2^]	-0.009	0.937	-0.015	0.914	-0.041	0.756

NYHA: New York Heart Association; H: healthy controls; CHF: chronic heart failure; CHF-II: CHF in NYHA class II; CHF-III: CHF in NYHA class III; I/E: ratio of the inspiratory phase duration to the expiratory one; RSA: respiratory sinus arrhythmia; *r*: Pearson product moment correlation coefficient; *p*: probability of type I error.

## Discussion

The main findings of this study can be summarized as follows: i) the HPVA was not observable in middle-aged H subjects during SB but it could be evoked via CB at 15 breaths/minute; ii) the HPVA was present neither in CHF patients during SB nor could be evoked via CB; iii) regardless of RSA levels, HPVA was not associated with the I/E ratio in both H and CHF patients; iv) in the presence of physiological I/E ratios (i.e. I/E<1), HPVA was not associated with RSA in both H and CHF patients.

### Impact of CB on HPVA of middle-aged H subjects

It is well-known that aging reduces HPVA [7, 10, 11]. This study confirms the negative impact of aging on HPVA [7, 10, 11]. Indeed, middle-aged H controls did not exhibit HPVA as suggested by the similar values of PI found in the original and surrogate HP series during SB. The loss of HPVA with age has been interpreted as a sign of the reduced complexity of the cardiac control during senescence [7, 10] that is commonly detected using different approaches such as conditional entropy or predictability [32–34]. This reduced complexity might be the result of the impairment of the central autonomic network responsible for the generation of low and high frequency rhythms at the level of the brainstem [35–37] and/or the consequence of the loss of peculiar characteristics of reflex circuits, such as the different baroreflex responses to positive and negative arterial pressure changes [7, 38]. Also the loss of sympathetic sinus node responsiveness with age [34, 39] might be responsible for the missing HPVA in middle-aged H subjects. Remarkably, CB at 15 breaths/minutes was still able to increase HPVA in middle-aged H subjects, thus indicating that HPVA can be manipulated by regularizing breathing at a well-tolerated rate. This result might indicate that respiration could stimulate via afferent pathways central respiratory pattern generators with the inherent possibility of driving asymmetric neural outflows directed to the heart [40, 41] and/or reflex circuits with asymmetric sensitivity, such as the baroreflex, through stronger periodic modifications of the venous return and stroke volume [42, 43] linked to a more profound breathing at a slower rate [44]. Respiratory-driven actions might unveil the asymmetric activity of some components of the cardiovascular neural control at central [35–37] and/or peripheral levels [7, 38].

### Impact of CB on HPVA of CHF patients

This study confirms the negative impact of pathological conditions on HPVA [8, 10–13]. Indeed, in keeping with previous results [8], CHF subjects did not exhibit HPVA, as suggested by the similar values of PI found in the original and surrogate HP series during SB. In addition, unlike H controls, CB could not induce HPVA. Since CHF is known to alter cardiovascular control [45–47], the lack of HPVA and the inability of CB to evoke HPVA might be taken as an additional sign of the derangement occurring at central and/or peripheral levels and a hallmark of the reduced complexity of the cardiac control in CHF [10]. However, other phenomena, such as the reduced sinus node responsiveness and saturation of cardiac receptors to neural inputs, typically observed in CHF patients, might play a role as well [34, 39, 45].

### HPVA was not associated with I/E ratio in both H and CHF patients

During SB we confirm that the EXP duration was longer than the INSP one in middle-aged H subjects, thus indicating the asymmetry of the respiratory pattern [17, 18]. As a consequence, regardless of the experimental condition, the I/E ratio was smaller than 1 in the H group [17, 18]. The asymmetry of the respiratory pattern was preserved in both CHF-II and CHF-III groups. During CB we observed that both EXP and INSP phase increased proportionally in response to the overall modification of respiratory period compared to SB. This conclusion held regardless of the group, thus leading to a constancy of the I/E ratio across experimental conditions and groups. Since tachycardic runs are more likely during INSP and the bradycardic ones are more frequent during EXP [23], it can be hypothesized a positive association between PI and I/E ratio. As a matter of fact, the transition from a physiological I/E ratio of 1:2 to an imposed and less physiological I/E ratio of 2:1 increased PI [17]. In the present study with physiological I/E ratios (i.e. I/E<1) and in presence of limited RSA, as it occurred in these middle-aged H subjects and CHF patients, we did not find any correlation between PI and I/E ratio. This result was confirmed regardless of the group. Thus, we conclude that the variability of I/E ratio about a physiological value cannot explain *per se* the HPVA phenomenon.

### In the presence of physiological I/E ratios, HPVA was not associated with RSA in both H and CHF patients

In the presence of physiological I/E ratios, i.e. I/E<1, and limited RSA, it was hypothesized that PI is about 50% and even less [7]. Furthermore, it was conjectured that, even in absence of an I/E ratio increase, the PI could migrate toward 50% and eventually overcome 50%, if RSA increases [7]. The significant increase of RSA in all groups during CB allowed us to test this hypothesis. Since no correlation was found between PI and RSA, the initial hypothesis was rejected. Remarkably, this conclusion held after pooling together data relevant to SB and CB, namely after a procedure that allowed us to span a larger RSA range due to the RSA increase during CB compared to SB. For example, in CHF patients RSA increased during CB, while PI remained unvaried. Therefore, the observed increase of PI in H individuals during CB should not be considered the trivial consequence of an increased vagal control responsible for an augmented RSA [48]. The uncorrelation between PI and RSA would be in agreement with the peripheral origin of the HPVA, namely the result of some asymmetric properties of reflex circuits [7, 38], rather than with the central one, namely the generation of an asymmetric periodical input from central pattern generators at brainstem level [35–37]. We conclude that PI and RSA carry non-redundant information in physiological conditions featuring values of the I/E ratio smaller than 1. Therefore, we recommend the exploitation of PI in any study based on HPV analysis as an additional marker able to point out a peculiar aspect of the cardiac control that is not fully addressed by other HPV markers.

### Potential clinical impact of HPVA assessment

HPVA analysis may be of clinical importance given the association between HPVA markers and cardiovascular aging [7, 10, 11]. Indeed, HPVA may provide further information about healthy aging, thereby opening the possibility of testing the efficacy of treatment strategies aimed at slowing the aging process. The complementarity of HPVA markers with respect to more traditional indexes of cardiac control derived from HPV (e.g. RSA), demonstrated in the present study, might provide further indications for the evaluation of the efficacy of countermeasures. Moreover, HPVA analysis may also be of clinical relevance owing to the link between HPVA markers and peculiar features of the baroreflex [7]. The indirect estimation of features linked to the different responses of HP to arterial pressure rises or falls based on HPVA markers might facilitate the detection of subjects at risk of impaired cardiac control responses to usual stressors (e.g. orthostatic challenges) and/or to particular conditions (e.g. post-exercise recovery).

## Conclusions

In the present study we investigated the relation between HPVA, as inferred from the percentage of negative HP changes (i.e. the PI), and RSA in middle-aged H controls and in CHF patients, both featuring physiological I/E ratios. We found that HPVA was uncorrelated with the RSA even when pooling together data relevant to SB and CB. Therefore, we conclude that RSA is not a determinant of HPVA in middle-aged H controls and CHF patients in presence of physiological values of the I/E ratio. This finding suggests that HPVA markers contain non-redundant information compared to more traditional HPV markers such as the RSA. Since HPVA increased during CB only in H subjects, we conclude that regularizing breathing at a well-tolerated rate (i.e. 15 breaths/minute) might stimulate efferent asymmetric autonomic patterns directed to the heart and/or might induce asymmetric responses of reflex cardiac control circuits such as the baroreflex. We promote the use of CB to modify HPVA in middle-aged H subjects and to stratify individuals according to the ability of CB to evoke HPVA. It remains to be elucidated whether the exploitation of HPVA markers could allow the exploration of peculiar aspects of the central autonomic network and/or peripheral reflex controls and whether information provided by HPVA markers could be of clinical value.

## Supporting information

S1 Dataset Database of the HPV and respiratory signal markers. It contains time domain, frequency domain and HPVA markers as well durations of the INSP and EXP phases and I/E ratio in all the groups (i.e. H, CHF-II and CHF-III) during SB and CB.
(XLSX)
